# Editorial: The Roles of GnIH in Reproductive Function and Behavior

**DOI:** 10.3389/fendo.2018.00019

**Published:** 2018-01-31

**Authors:** Takayoshi Ubuka, Ishwar Parhar, Lance J. Kriegsfeld, Kazuyoshi Tsutsui

**Affiliations:** ^1^Jeffrey Cheah School of Medicine and Health Sciences, Brain Research Institute Monash Sunway, Monash University Malaysia, Petaling Jaya, Malaysia; ^2^Department of Psychology, Helen Wills Neuroscience Institute, University of California at Berkeley, Berkeley, CA, United States; ^3^Laboratory of Integrative Brain Sciences, Department of Biology, Center for Medical Life Science, Waseda University, Tokyo, Japan

**Keywords:** gonadotropin-inhibitory hormone, RFamide-related peptide, LPXRF-amide peptide family, Reproduction, hypothalamic–pituitary–gonadal axis

**Editorial on the Research Topic**

**The Roles of GnIH in Reproductive Function and Behavior**

Since the discovery of gonadotropin-releasing hormone (GnRH) at the beginning of 1970s, it has been believed that GnRH is the only hypothalamic neuropeptide that regulates gonadotropin release in vertebrates. In 2000, however, a novel hypothalamic neuropeptide that actively inhibits gonadotropin release was discovered in quail and termed gonadotropin-inhibitory hormone [GnIH, (1)]. GnIH is one of the RFamide peptides, which is also known as RFamide-related peptide (RFRP) in mammals. Following the discovery, the next 17 years of research revealed that GnIH is highly conserved across vertebrates including humans, and GnIH is involved in a number of physiological and behavioral functions related to reproduction (2–4). This research topic compiles original research and review articles describing the discovery and progress of GnIH research from leading scientists in the field.

The first review paper by Tsutsui et al. (2) describes how they discovered GnIH from the quail brain and showed its inhibitory effect on gonadotropin synthesis and release (Tsutsui et al.). They also describe studies over the past decade and a half that established the physiological function of GnIH as a key player regulating reproduction across vertebrates by acting on the brain and pituitary to modulate their reproductive physiology and behavior. They further introduce recent evidence indicating that GnIH regulates reproductive behavior through changes in neuroestrogen biosynthesis in the brain (5).

The review by Leon and Tena-Sempere describes their investigations on how GnIH (RFRP) acts centrally to suppress GnRH/gonadotropin secretion directly or indirectly cooperating with other stimulatory inputs such as kisspeptin in the dynamic regulation of the hypothalamic–pituitary–gonadal (HPG) axis. They focus on studies using pharmacological tools and functional genomics in rodent models. In 2006, RF9 was invented as a potent and selective antagonist of neuropeptide FF (NPFF) receptors including GnIH receptor (GPR147), which is also known as NPFF1 (6). Tena-Sempere’s group was the first to show that central administration of RF9 evokes robust luteinizing hormone (LH) secretory responses in rats (7). They were also the first to create an NPFF1 null mouse (8). Although NPFF1 null mouse preserved pubertal progression and fertility, a rapid drop of LH was not observed in NPFF1 null mouse by food deprivation (8) suggesting the role of GnIH in the regulation of feeding (9) and stress (10).

Poling and Kauffman discuss the regulation of GnIH (RFRP-3) neurons by sex steroids and leptin during development and sexual maturation in rodents. They highlight significant changes in GnIH expression and neuronal activation during postnatal and pubertal development and discuss the role of GnIH receptors (GPR147; NPFF1) for normal pubertal timing and developmental LH secretion based on their studies (11). Geraghty et al. report their investigation on the role of GnIH (RFRP) and GnIH receptor (GPR147; NPFF1) in the regulation of age-related reproductive decline in female rats. Interestingly, females exhibited an increase in GnIH (RFRP) and GPR147 (NPFF1) mRNA expression in the hypothalamus before irregular estrous cycle in middle age rats. This transient increase was followed by subsequent decreases in kisspeptin and GnRH mRNA expression. Expression of GnIH (RFRP) and GPR147 (NPFF1) also increased in the ovaries with advancing age (Geraghty et al.). The results suggest a novel role of GnIH (RFRP) signaling system in the regulation of reproductive cessation.

Reproduction is regulated seasonally in species living in temperate zones and GnIHs (RFRP-1 and -3) are believed to play a critical role in the central control of seasonal reproduction. Henningsen et al. summarize the role and underlying mechanisms of GnIH (RFRP) in the seasonal control of reproduction, primarily focusing on mammalian species. In mammals, the GnIH (RFRP) system is persistently suppressed by short photoperiod and melatonin (12), which is opposite to quail (13). Central chronic administration of GnIH (RFRP-3) in short day-adapted male Syrian hamsters fully reactivates the reproductive axis (14), highlighting the importance of the seasonal changes in GnIH (RFRP) in proper regulation of the reproductive axis. Anjum et al. propose GnIH (RFRP) as a mediator between adiposity and impaired testicular function in their original research article. GnIH (RFRP) treatment increased food intake, upregulation of glucose transporter 4, and increased uptake of triglycerides in the adipose tissue of mice. On the other hand, treatment with GnIH (RFRP) decreased glucose uptake by downregulation of glucose transporter 8 expression and decreased testosterone synthesis. GnIH (RFRP) treatment also showed decreased expression of insulin receptor in the testis. Their findings suggest a new role of GnIH (RFRP) in fat accumulation, in addition to negative regulation of testosterone synthesis [Anjum et al.; (15)].

Various stressors suppress the HPG axis and consequently induce reproductive dysfunction. Iwasa et al. review the role of GnIH (RFRP) in stress-induced reproductive dysfunction. Psychological and immune stress increase GnIH (RFRP) expression and suppresses GnRH and gonadotropin secretion (10, 16). It was shown that glucocorticoid acts as a mediator of stress and GnIH (RFRP) (17). Soga et al. investigated the effect of early-life social isolation on serotonergic and GnIH neuronal system using enhanced green fluorescent protein (EGFP)-tagged GnIH transgenic rats (18) in their research article. They found that the total number of EGFP-GnIH neurons in socially isolated rats was the same as control rats, but c-Fos expression in GnIH neurons was significantly reduced in socially isolated rats. Serotonin fiber juxtapositions on EGFP-GnIH neurons were also reduced in socially isolated rats. Teo et al. found in their original research that socially isolated rats display greater CLOCK expression in the dark phase, while control rats display increased CLOCK expression in the light phase in EGFP-GnIH neurons in the dorsomedial hypothalamus. They also found that β-catenin expression pattern was disrupted in GnIH cells by social isolation (Teo et al.).

Fish represent more than the half of recognized living vertebrate species. Fish also include model species with scientific, clinical, and economic importance. Muñoz-Cueto et al. summarize all GnIH precursor and peptide sequences identified in fish, distribution of GnIH and GnIH receptor (GPR147; NPFF1) in central and peripheral tissues, physiological actions of GnIH on the HPG axis, as well as other reported effects of GnIH, and regulatory mechanisms of GnIH in fish. Finally, Ubuka and Parhar highlight the stimulatory effect of GnIH in the HPG axis, which was shown in mammals and in fish (2, 3, 12, 14) and investigate their pharmacological and physiological mechanisms as a perspective of future research.

## Author Contributions

TU wrote the manuscript. IP, LJK, and KT edited the manuscript.

## Conflict of Interest Statement

The authors declare that the research was conducted in the absence of any commercial or financial relationships that could be construed as a potential conflict of interest.
